# Molecular Identification of Rare Clinical Mycobacteria by Application of 16S-23S Spacer Region Sequencing

**Published:** 2012

**Authors:** Hasan Shojaei, Hashemi Abodolrazagh, Parvin Heidarieh, Fazel Pourahmad, Daei Naser Daei Naser

**Affiliations:** 1 *Department of Microbiology, School of Medicine, Isfahan University of Medical Sciences, Isfahan, Iran*; 2 *Infectious and Tropical Disease Research Center, Ahvaz Jundishapur University of Medical Sciences, Iran*; 3 *Microbiology Group, Department of Pathobiology, School of Public Health, Tehran University of Medical Sciences, Tehran, Iran*; 4 *School of Veterinary Medicine, Ilam University, Ilam, Iran*; 5 *Infectious Diseases and Tropical Medicine Research Center, Isfahan University of Medical Sciences, Isfahan, Iran*

**Keywords:** Internal Transcribed Spacer, Mycobacterium, Sequence Analysis

## Abstract

**Objective(s):**

In addition to several molecular methods and in particular 16S rDNA analysis, the application of a more discriminatory genetic marker, i.e., 16S-23S internal transcribed spacer gene sequence has had a great impact on identification and classification of mycobacteria. In the current study we aimed to apply this sequencing power to conclusive identification of some Iranian clinical strains of mycobacteria.

**Materials and Methods:**

The test strains consisted of nineteen mycobacterial isolates which were initially identified by the use of conventional phenotypic techniques and molecular methods and subjected to further definitive identification using the 16S-23S internal transcribed spacer gene sequencing.

**Results:**

Out of 19 studied strains, 7 isolates were found to be rapidly growing and 12 isolates as slowly growing mycobacteria. With the exception of one isolate, i.e., the isolate HNTM87, which yielded a distinct ITS sequence incomparable with all previously identified mycobacteria, the remaining isolates produced the sequences similar to the established mycobacteria and were clearly identified and differentiated from closely related taxa. A phylogenetic tree based on maximum parsimony analysis of 16S-23S internal transcribed spacer gene sequences constructed showing the relatedness of Iranian clinical isolates with the closely related type species of mycobacteria.

**Conclusion:**

This study showed that the 16S-23S internal transcribed spacer gene of the genus *Mycobacterium* exhibits a high variation which is of value for discriminating closely related taxa and could be used independently or in combination with 16S rDNA sequencing to delineate the true identity of rare mycobacterial species.

## Introduction

To date, the genus *Mycobacterium* comprises over 150 species (http://www.bacterio.cict.fr/). Several species other than *M. tuberculosis* which are called nontuberculous mycobacteria (NTMs) are becoming increasingly recognized as significant human pathogens (1).

In the last decade, the rapid development of molecular techniques has led to a great increase in our knowledge of mycobacterial identification and taxonomy (2). Methods for the detection and identification of mycobacteria include nucleic acid probes (3), PCR hybridization with species-specific probes (4, 5), PCR RFLP analysis (6, 7) and nucleic acid sequencing (8).

In recent years a well-established sequencing analysis using 16S rRNA gene (rDNA) has greatly contributed to an accurate identification and classification of mycobacteria (8). However, some members of clinically significant mycobacterial complex groups e.g., *M. kansasii *complex (*M. kansasii* and *M. gastri*), *M. fortuitum* complex (*M. fortuitum*, *M. chelonae *and* M. abscessus*), *M. avium *complex (*M. avium *and *M.* *interacellurae*), *M. farcinogenes* complex (*M. farcinogenes *and* M. senegalense*) and *M. terrae *complex *(M terrae, M nonchromogenicum, *and* M triviale) *are difficult to be differentiated from each other due to a very high degree of 16S rDNA sequence similarity (9, 10).

Several studies have shown that sequencing of 16S-23S internal transcribed spacer (ITS) gene could help to differentiate and identify the closely related mycobacterial species (11-13). In the current study we aimed to apply this sequencing power to the precise diagnosis of clinical strains of mycobacterial species which were difficult to correctly delineate their identity by conventional microbiological testing or even 16S rDNA sequencing.

## Materials and Methods

***Isolates***

Mycobacterial strains investigated in the present study included 19 clinical strains which had been isolated in or sent to our laboratory, the Molecular Laboratory of Clinical and Environmental Mycobacteria at *Infectious Diseases and Tropical Medicine Research Center, Isfahan*, Isfahan, Iran.

***Initial identification and classification***

The isolates were initially screened by the main conventional phenotypic tests including standard morphological and biochemical assays previously established for identification of mycobacteria (14). The preliminary identification was confirmed by the key molecular markers including a genus specific segment of 65-kDa heat shock protein gene (*hsp*) and 16S rDNA sequencing following standard procedures (15,16). 

***Molecular identification by ITS gene sequencing***

All isolates which were preliminarily identified and classified into the previously known mycobacterial clusters according to phenotypic and molecular features (Table 1) were subjected to more definitive identification by ITS gene sequencing following the procedures described herein.

***DNA extraction and purification***

Chromosomal DNA was extracted using a method of Pitcher *et al* (17) with a slight modification to facilitate the susceptibility of cells to the standard digestion (16). In brief, after thermal inactivation, a pretreatment of biomass with lipase (Type VII; final concentration, 2 mg/ml [Sigma]) and a further treatment with proteinase K (100 pg/ml) and sodium dodecyl sulfate (final concentration, 0.5% [wt/vol]) were applied. The DNA was purified by phenol chloroform-isoamyl alcohol and precipitated with isopropanol. The precipitate was washed in 70% ethanol, dehydrated and dissolved in 100 µl of Milli-Q water and stored in 20 °C freezer until use.

***Amplification of ITS gene from clinical isolates of NTMs***

Amplification of the full ITS segment was performed with primers (16S-1511f-5’AAGTCGTAACAAGGARCCG -3’ and 23S-23r-5’ YYGCCAAGGCATCCACC-3’) in a 50 µl reaction mixture containing 10 mM Tris-HCl (PH 8.3), 50 mM KCl, 1.5 mM MgCl2, 200 µM (each) deoxynucleoside triphosphate (dATP, dGTP, dCTP, and dUTP), 10 pmol of each primer, 0.5 U of Taq DNA polymerase, and 5 µl (50 ng) of extracted DNA (11). The thermal profile involved initial denaturation for five min at 95 °C and thirty cycles with the following steps: 30 sec of denaturation at 94 °C, 30 sec of annealing at 55 °C, and one min of extension at 72 °C and a final extension of 10 min at 72 °C (11). 

***Nucleotide sequencing***

The amplified ITS gene generated by PCR reaction was purified using a QIA quick PCR purification kit (Qiagen, Chatsworth, Calif) as described in the instructions of the manufacturer. The purified PCR products were directly sequenced with the forward 16S-1511f and reverse 23S-23r primers using an ABI 3100 genetic analyzer and a Big Dye Terminator cycle sequencing kit by SEQLAB Company (Germany). 

***Data analysis of ITS gene sequences***

The obtained sequences were aligned with the published ITS gene sequences of the type strains of mycobacteria (retrieved from GenBank^TM^ database) using the jPhydit software package according to primary-structure (18). Comparative analyses of ITS gene sequences were performed with distance matrix, maximum-parsimony, and maximum-likelihood methods (19) as implemented in the Mega4 program (20). Tree topologies were tested by bootstrap analysis on 1000 resampling (21). 

***Nucleotide sequence accession numbers***

The GenBank accession numbers of the ITS gene for mycobacterial isolates determined in this work are: HQ406781-98 and HM536981.

## Results

On the basis of growth characteristics (Table 1), 7 isolates were rapidly growing mycobacteria (RGM) and the remaining 12 clinical isolates were slowly growing mycobacteria (SGM). The isolates were initially classified into four Runyon's groups (22) and further speciated by 16S rDNA sequencing (Table 1).

PCR amplification of the ITS region with the primers 16S-1511f and 23S-23r resulted in detection of a single band of approximately 550 bp and 400 bp for RGM and SGM respectively. The variation in product length was considerable between slow and rapid growing mycobacteria showing that the ITS gene of the SGM are shorter than that of RGM. This is due to the features that clearly differentiate SGM from RGM on the basis of PCR product of ITS gene. 

With the exception of HNTM87 which yielded a distinct ITS gene sequence which initially failed to produce any significant matches to sequences within genus* Mycobacterium*, the remaining isolates produced sequences that were clearly identified and differentiated from closely related taxa (Table 1, Figure 1). These are the isolates HNTM19 and HNTM20 as *M. avium* and *M. interacellurae*, the isolate HNTM2 as *M. Kansasii*, the isolate HNTM95 as *M. fortuitum*, the isolate HNTM71 as *M. porcinum *and the isolates HNTM70, HNTM80 and M137 as *M. conceptionense* (Table 1). The ITS gene sequence analysis reconfirmed the identity of the remaining isolates i.e., HNTM11, HNTM64, HNTM65, HNTM75, HNTM79, HNTM83, HNTM92, HNTM104 and HNTM105 which were initially well identified by 16S rDNA sequencing (Table 1).

**Figure 2:**
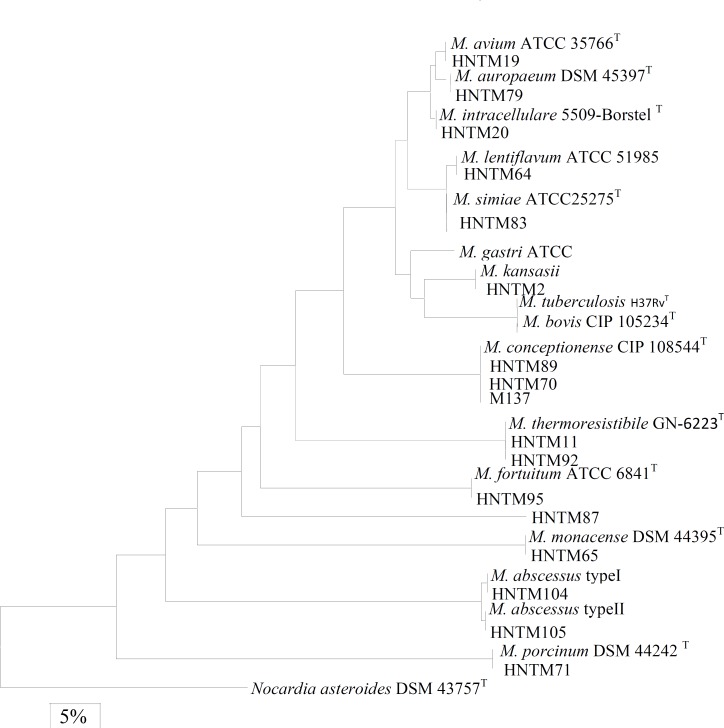
Phylogenetic tree showing the divergence of ITS gene sequences of the investigated mycobacteria. The topology of the tree was evaluated according 1000 resampling of sequences in bootstrap analysis (21). The corresponding sequence of the *Nocardia asteroides* DSM 43757^T^ was used as an outgroup reference. The bar represents 5% estimated sequence divergence

The isolate HNTM72 which was placed within NTM due to a negative niacin test found to be a member of *M. tuberculosis* complex (MTBC) by 16S rDNA sequencing. This was reconfirmed by ITS gene sequencing. A Phylogenetic tree based on maximum parsimony analysis of the ITS gene sequences was constructed to find the relatedness of Iranian clinical isolates with the closely related type species of myco+6666666bacteria (Figure 2). The resulting dendrogram showed that the slowly and rapidly growing isolates were clearly grouped into two separate clusters.

**Table 1:** Identification of Iranian clinical isolates of NTMs based on ITS gene sequence analysis

Isolates	Runyon's groups	Species by 16S rDNA sequence analysis	Species by ITS sequence analysis	Differences /Total (base pair)	Similarity (%)
HNTM2	I	*M. kansasii-gastri complex*	*M. kansasii*	0/275	100
HNTM11	IV	*M. thermoresistibile*	*M. thermoresistibile*	0/296	100
HNTM19	III	MAC	*M. avium*	0/274	100
HNTM20	III	MAC	*M. intracellulare*	0/279	100
HNTM64	II	*M. lentiflavum*	*M. lentiflavum*	0/280	100
HNTM65	IV	*M. monacense*	*M. monacense*	3/313	99.04
HNTM70	IV	*M. fortuitum complex*	*M. conceptionense*	0/234	100
HNTM71	IV	*M. fortuitum complex*	*M. porcinum*	0/287	100
HNTM72	III	MTBC	MTBC	0/266	100
HNTM75	II	*M. simiae*	*M. simiae*	0/282	100
HNTM79	II	*M. auropaeum*	*M. auropaeum*	2/259	99.02
HNTM83	II	*M. simiae*	*M. simiae*	0/282	100
HNTM87	IV	*Mycobacterium sp.*	*Mycobacterium sp.*	79/280	40
HNTM89	IV	*M. fortuitum *complex	*M. conceptionense*	0/234	100
HNTM92	IV	*M. thermoresistibile*	*M. thermoresistibile*	0/296	100
HNTM104	IV	*M. abscessus*	*M. abscessus*	0/247	100
HNTM105	IV	*M. abscessus*	*M. abscessus*	0/248	100
HNTM95	IV	*M. fortuitum *complex	*M. fortuitum*	0/301	100
M137	IV	*M. fortuitum *complex	*M. conceptionense*	0/234	100

MAC; Mycobacterium avium complex, MTBC; Mycobacterium *tuberculosis* complex

## Discussion

Identification of mycobacterial species by conventional methods including growth characteristics and biochemical tests are time-consuming and often not unequivocal in their interpretation (2, 8). Currently, analysis of the gene encoding 16S rDNA is widely accepted for identification of mycobacterial isolates to the species level; however this technique is unable to differentiate members of several closely related species called complex groups (5, 7, 9-11).

In the current study we intended to use a more discriminatory DNA sequencing technology, i.e., the ITS gene sequence analysis, to specify the exact identity of some Iranian clinical isolates. These isolates were formerly classified as a member of mycobacterial complex groups or their identity was yet to be resolved more definitely or reconfirmed due to ambiguity in a few phenotypic and biochemical features. By using ITS sequencing, these strains showed a significant nucleotide differences with the closely related taxa. Furthermore, they formed a distinct node in phylogenetic tree representing a previously defined species. With 16S rDNA analysis these isolates had shown a high nucleotide similarity with the closely related mycobacteria which in turn resulted in the formation of a joint line of descent in phylogenetic analysis.

**Figure 1:** Alignment of selected positions of ITS gene sequences including those of 19 Iranian clinical isolates of mycobacteria investigated in this study together with sequences of closely related species.

Taxon	1	47	50	51	53	54	56	58	68	70	71	72	208	209	210	211	227	230	239	240	241	249	250	251	252	253	254	255	256	257	258	259	260	261
*M. avium* (ATCC 35766^T^)	A	-	G	C	-	G	A	C	T	-	C	C	T	-	-	-	-	-	T	G	G	-	T	C	C	C	T	C	C	A	T	-	-	C
*M. abscessus* genotype typeII	.	-	A	A	A	.	.	G	.	-	G	A	C	-	T	G	-	T	A	C	.	G	.	.	A	.	C	.	T	G	-	-	-	.
*M. abscessus* genotype typeI	.	-	A	A	A	.	.	G	.	-	G	G	C	-	T	G	-	T	A	C	.	G	.	.	A	.	C	.	T	G	-	-	-	.
*M. auropaeum* (DSM 45397^T^)	.	-	.	.	-	.	.	.	.	-	.	.	-	-	T	.	-	-	G	T	.	-	.	.	.	.	.	.	.	.	.	-	-	.
*M. conceptionense* (CIP108544^T^)	.	G	.	-	-	T	.	.	.	-	-	.	-	-	-	.	-	-	.	T	.	-	.	.	.	.	A	.	.	G	C	-	-	.
*M. fortuitum*	.	C	C	-	-	.	.	.	C	-	G	G	-	-	T	G	-	-	-	-	-	-	-	.	.	.	.	-	-	-	-	-	-	T
*M. gastri *(ATCC 15754^T)^	.	-	.	T	-	A	.	.	C	-	.	.	-	-	T	.	C	-	.	T	.	-	.	.	.	.	A	.	.	.	.	-	-	.
*M. intracellulare* (5509 Borstel^T)^	.	-	.	.	-	.	.	.	.	-	.	.	-	-	T	.	-	-	G	A	.	-	.	.	.	.	.	.	.	.	.	-	-	.
*M. kansasii*	.	-	.	.	-	A	.	.	.	-	.	T	-	-	T	T	-	-	.	T	.	-	.	.	.	.	A	.	.	.	.	-	-	.
*M. lentiflavum*	.	-	.	T	-	.	.	.	.	-	.	T	-	C	T	T	-	-	-	-	-	-	-	.	.	.	.	-	-	-	-	-	-	T
*M. monacense*	.	G	T	.	-	.	T	A	A	T	.	G	-	G	G	G	C	A	G	C	.	-	.	.	A	.	.	.	.	C	.	-	-	T
*M. porcinum* (DSM 44242^T^)	.	G	C	G	-	T	G	.	G	-	T	G	A	C	A	.	C	-	G	C	.	-	.	.	A	.	.	.	.	C	.	-	-	T
*M. simiae *(ATCC 25275^T^)	.	-	.	T	-	.	.	.	.	-	.	T	-	C	T	T	-	-	.	.	A	-	G	A	.	A	A	.	A	G	G	-	-	.
*M. thermoresistibile* (GN-6223)	.	-	T	G	-	.	T	.	.	-	G	G	-	-	G	.	-	-	G	T	.	-	.	.	.	.	.	.	.	.	.	-	-	.
*M. tuberculosis* (H37Rv)	.	-	.	T	-	A	G	.	.	-	.	T	-	-	T	.	-	-	.	T	.	-	.	.	.	.	A	.	.	.	.	-	-	.
HNTM2	.	-	.	.	-	A	.	.	.	-	.	T	-	-	T	T	-	-	.	T	.	-	.	.	.	.	A	.	.	.	.	-	-	.
HNTM11	.	-	T	G	-	.	T	.	.	-	G	G	-	-	G	.	-	-	G	T	.	-	.	.	.	.	.	.	.	.	.	-	-	.
HNTM19	.	-	.	.	-	.	.	.	.	-	.	.	-	-	-	.	-	-	.	.	.	-	.	.	.	.	.	.	.	.	.	-	-	.
HNTM20	.	-	.	.	-	.	.	.	.	-	.	.	-	-	T	.	-	-	G	A	.	-	.	.	.	.	.	.	.	.	.	-	-	.
HNTM64	.	-	.	T	-	.	.	.	.	-	.	T	-	C	T	T	-	-	-	-	-	-	-	.	.	.	.	-	-	-	-	-	-	T
HNTM65	.	G	T	.	-	.	T	A	A	T	.	G	-	G	G	G	C	A	G	C	.	-	.	.	A	.	.	.	.	C	.	-	-	T
HNTM70	.	G	.	-	-	T	.	.	.	-	-	.	-	-	-	.	-	-	.	T	.	-	.	.	.	.	A	.	.	G	C	-	-	.
HNTM71	.	G	C	G	-	T	G	.	G	-	T	G	A	C	A	.	C	-	G	C	.	-	.	.	A	.	.	.	.	C	.	-	-	T
HNTM72	-	-	.	T	-	A	G	.	.	-	.	T	-	-	T	.	-	-	.	T	.	-	.	.	.	.	A	.	.	.	.	-	-	.
HNTM75	.	-	.	T	-	.	.	.	.	-	.	T	-	C	T	T	-	-	.	.	A	-	G	A	.	A	A	.	A	G	G	-	-	.
HNTM79	.	-	.	.	-	.	.	.	.	-	.	.	-	-	T	.	-	-	G	T	.	-	.	.	.	.	.	.	.	.	.	-	-	.
HNTM83	.	-	.	T	-	.	.	.	.	-	.	T	-	C	T	T	-	-	.	.	A	-	G	A	.	A	A	.	A	G	G	-	-	.
HNTM87	-	-	C	G	-	.	.	-	.	-	G	T	-	C	T	T	-	-	G	T	.	-	G	.	-	-	.	.	.	G	C	-	-	G
HNTM89	.	G	.	-	-	T	.	.	.	-	-	.	-	-	-	.	-	-	.	T	.	-	.	.	.	.	A	.	.	G	C	-	-	.
HNTM92	.	-	T	G	-	.	T	.	.	-	G	G	-	-	G	.	-	-	G	T	.	-	.	.	.	.	.	.	.	.	.	-	-	.
HNTM95	.	C	C	-	-	.	.	.	C	-	G	G	-	-	T	G	-	-	-	-	-	-	-	.	.	.	.	-	-	-	-	-	-	T
HNTM104	.	-	A	A	A	.	.	G	.	-	G	A	C	-	T	G	-	T	A	C	.	G	.	.	A	.	C	.	T	G	-	-	-	.
HNTM105	.	-	A	A	A	.	.	G	.	-	G	G	C	-	T	G	-	T	A	C	.	G	.	.	A	.	C	.	T	G	-	-	-	.
M137	.	G	.	-	-	T	.	.	.	-	-	.	-	-	-	.	-	-	.	T	.	-	.	.	.	.	A	.	.	G	C	-	-	.
																									

## Conclusion

Our study revealed that the ITS gene of the genus *Mycobacterium* exhibits a high variation which is of value for discriminating closely related taxa. However, in terms of MTBC, this is not the case since the technique lacks a sufficient power to differentiate and separate members of the group from each other.
